# Downregulation of HuR as a new mechanism of doxorubicin resistance in breast cancer cells

**DOI:** 10.1186/1476-4598-11-13

**Published:** 2012-03-21

**Authors:** Elisa Latorre, Toma Tebaldi, Gabriella Viero, Antonino Maria Spartà, Alessandro Quattrone, Alessandro Provenzani

**Affiliations:** 1 Laboratory of Genomic Screening, Centre for Integrative Biology, CIBIO, University of Trento, Trento, Italy; 2 Laboratory of Translational Genomics, Centre for Integrative Biology, CIBIO, University of Trento, Trento, Italy; 3 CNR Institute of Biophysics, Povo, Trento, Italy

**Keywords:** HuR, Doxorubicin, Drug resistance, Apoptosis, Translational regulation

## Abstract

**Background:**

HuR, an RNA binding protein involved in the post-transcriptional regulation of a wide spectrum of mRNAs, has been demonstrated to be a determinant of carcinogenesis and tumor aggressiveness in several cancer types. In this study, we investigated the role of HuR in the apoptosis and in the chemoresistance induced by the widely used anticancer drug doxorubicin in human breast cancer cells (MCF-7).

**Results:**

We showed that HuR acts in the early phase of cell response to doxorubicin, being induced to translocate into the cytoplasm upon phosphorylation. Reducing HuR levels diminished the apoptotic response to doxorubicin. Doxorubicin-induced apoptosis was also correlated with the presence of HuR in the cytoplasm. Rottlerin, which was able to block HuR nuclear export, had correspondingly antagonistic effects with doxorubicin on cell toxicity. The proapoptotic activity of HuR was not due to cleavage to an active form, as was previously reported. In *in vitro *selected doxorubicin resistant MCF-7 cells (MCF-7/doxoR) overexpressing the multidrug resistance (MDR) related ABCG2 transporter, we observed a significant HuR downregulation that was paralleled by a corresponding downregulation of HuR targets and by loss of rottlerin toxicity. Restoration of HuR expression in these cells resensitized MCF-7/doxoR cells to doxorubicin, reactivating the apoptotic response.

**Conclusions:**

The present study shows that HuR is necessary to elicit the apoptotic cell response to doxorubicin and that restoration of HuR expression in resistant cells resensitizes them to the action of this drug, thereby identifying HuR as a key protein in doxorubicin pharmacology.

## Background

Insurgence of drug resistance during chemotherapy is a major cause of cancer relapse and consequent failure of therapy for cancer patients. Genetic and epigenetic changes, resulting in gene expression reprogramming, play a major role in allowing adaptation to the presence of anticancer drugs [1]. One of the most important aspects of this phenomenon is the development of resistance and cross resistance to drugs having a mechanism of action unrelated to the single chemotherapeutic agent originally causing resistance, i.e. the MultiDrug Resistance phenotype (MDR) [2]. Resistance mechanisms are extremely complex, changing according to the type of drug that was used in therapy and spanning from the overexpression of drug extrusion pumps, as in the case of several cytotoxic compounds [3], to mutations or overexpression of the pharmacological target, as in the case of receptor tyrosine kinase inhibitors [4]. In the case of doxorubicin (doxo), a widely used chemotherapeutic agent, different mechanisms responsible for the onset of a drug resistant phenotype in cancer cell models have been recognized. The most common is characterized by enhanced expression of the P-glycoprotein, ABCB1 [5], a transmembrane pump responsible for drug efflux from cells. P-glycoprotein belongs to the family of ATP binding-cassette (ABC) transporters. Another member of this family, ABCG2, was more recently identified as involved in drug resistance to doxo as well [6]. The expression level of topoisomerase II [7,8], the molecular target of doxo, is another major factor implicated in doxo pharmacoresistance. Since doxo stimulates cell apoptosis through inhibition of topoisomerase II and consequent DNA damage, cells develop resistance by downregulating this enzyme [9].

Translational control is recognized as an increasingly important level of regulation of gene expression [10], but its impact in drug resistance has not yet been addressed fully. Among the major agents involved in translational control, the RNA binding protein (RBP) HuR is a pleiotropic protein [11] regulating many physiological processes. HuR acts as a mRNA stabilizer and/or a translational enhancer that binds to a large number of AU-rich element (ARE) containing mRNAs [12,13]. Many of the genes controlled by HuR are implicated in important physiological functions, such as embryonic development [14,15] and cell differentiation [16]. HuR overexpression or preferential cytoplasmic localization has been correlated with carcinogenesis in tissue biopsies and in cell models [17-19] and patient negative prognosis [20]. A caspase-truncated form of HuR has also been identified as a promoter of cell death [21,22].

In this work we explored the possibility that the involvement of HuR in the apoptotic response could contribute to the development of the resistance phenotype. First we show that HuR undergoes cytoplasmic translocation in MCF-7 cells exposed to doxo, and that this translocation is necessary to the doxo-induced triggering of apoptosis. We finally show that restoration of HuR expression in doxo-resistant, HuR-downregulating MDR cells is sufficient to reacquire sensitivity to this anticancer drug.

## Results

### Doxorubicin induces HuR phosphorylation and nucleocytoplasmic shuttling

**Figure 1:**
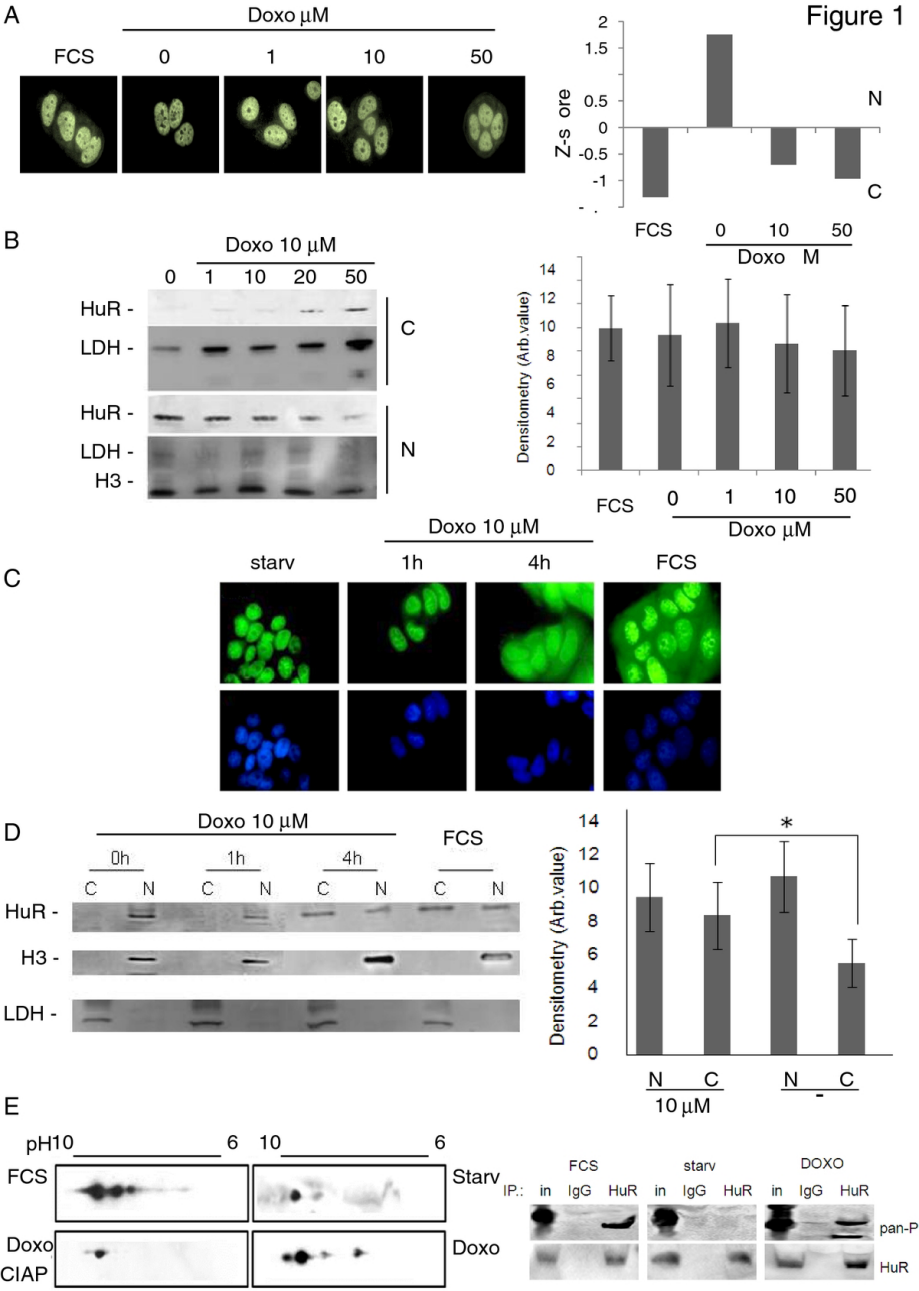
**A: HuR translocates from the nucleus to the cytosol after doxorubicin treatment**. Immunofluorescence (HuR in green, counterstaining DAPI in blue) on MCF-7 cells starved for 24 h and treated at increasing doxo concentration (from 1 to 50 μM) for 4 h. Quantification of HuR cytoplasmic translocation measured on immunofluorescence images by calculating the ratio of intensity signal in the nucleus and in the cytoplasm. An average of 300 cells for each experimental conditions were used. Z-score below zero indicates nuclear localization, above zero cytoplasmic localization. **B**: **Subcellular fractionation on increasing doses of doxorubicin and quantification of HuR expression level**. Western blotting on MCF-7 cells starved for 24 h and treated at increasing doxorubicin concentration (from 1 to 50 μM). The filter was probed with anti-H3 as nuclear fraction marker and anti-LDH as a cytosolic fraction marker. Quantification of total HuR protein measured by densitometric analyses on three independent western blots. **C**: **HuR translocates from the nucleus to the cytosol after 1 and 4 h doxorubicin treatment**. Immunofluorescence (HuR in green, counterstaining DAPI in blue) on MCF-7 cells starved or not (FCS) for 24 h and treated with doxorubicin 10 μM for 1 and 4 h. **D**: **Subcellular fractionation after 1 and 4 h doxorubicin treatment**. Western blotting on MCF-7 cells starved or not (FCS) for 24 h and with doxorubicin 10 μM for 1 and 4 h. The filter was probed with anti-H3 as nuclear fraction marker and anti-LDH as a cytosolic fraction marker. Quantification of HuR protein translocation after 10 μM doxo for 4 h measured by densitometric analyses on three independent western blots. * *p*-value < 0.01 with respect to starved conditions. **E: Doxorubicin induces modification in HuR phosphorylation**. 2D western blotting on whole cell lysates. MCF-7 cells were grown in standard condition (FCS), starved or in the presence of doxorubicin 10 μM for 4 h (doxo). As negative control for phosphorylation the doxorubicin sample was treated with calf intestinal alkaline phosphatase (CIAP). HuR immunoprecipitation blotted with anti HuR and pan anti-phospo antibody. As negative control IgG immunoprecipitation was blotted with anti HuR and pan anti-phospo antibodies. In: Input material, IgG: IgG immunoprecipitated material, HuR: anti-HuR immunoprecipitated material.

Since HuR is induced to relocate from the nucleus to the cytoplasm following DNA damaging stimuli such as UVR [23], we reasoned that an anticancer agent known to induce DNA damage as doxorubicin (doxo) could produce a similar effect. We starved MCF-7 cells for 24 h in order to induce nuclear localization of HuR (Figure 1A, B) [24]. Indeed, after 4 h of doxo addition, HuR translocated into the cytoplasm. The translocation effect was proportional to the applied dose, as quantified by calculating the ratio of the signal intensity of the protein in the nucleus versus the cytoplasm (Figure 1A). The total amount of HuR inside the cells did not change after doxo administration, as measured by densitometric analysis of three independent western blots (Figure 1B). As can be seen in Figure 1C and 1D, HuR began to accumulate in the cytoplasm after 1 h of 10 μM doxo addition. After 4 h, a two fold enrichment of the proteins was observed in the cytoplasm over the control condition (Figure 1D). Moreover, within the time frame of the experiment and notwithstanding the known cell damage induced by doxo that can result in the potential loss of nucleocytoplasmic compartmentalization, the nuclear membrane was still intact since nuclear and cytoplasmic markers (H3 and LDH, respectively) were clearly confined in their compartments while HuR accumulated in the cytoplasm. Since HuR shuttling is the consequence of post-translational modifications, including phosphorylation [25,26] we evaluated if doxo induced HuR phosphorylation. Lysates of cells treated with doxo resulted in the migration of HuR in a 2D Western blot stained with anti-HuR antibody at pH values lower than the pI of the native protein, which suggested that a series of phosphorylation events may have occurred after treatment with the drug. The bands were no longer visible after treatment of the lysates with alkaline phosphatases, consistent with the presence of phosphoryl groups (Figure 1E). This result was confirmed by immunoprecipitating HuR under the same experimental conditions and blotting with anti pan Ser/Thr antibody. A phosphorylation band was observed in the control reaction, i.e. in the presence of the serum, was absent during starvation, and reappeared after doxo administration. These findings suggest that doxo induces phosphorylation of HuR and accumulation of HuR in the cytoplasm, as is often observed with other DNA damaging treatment such as cisplatin [27].

### Apoptosis by doxorubicin is dependent on HuR phospohorylation and cytoplasmic translocation

**Figure 2:**
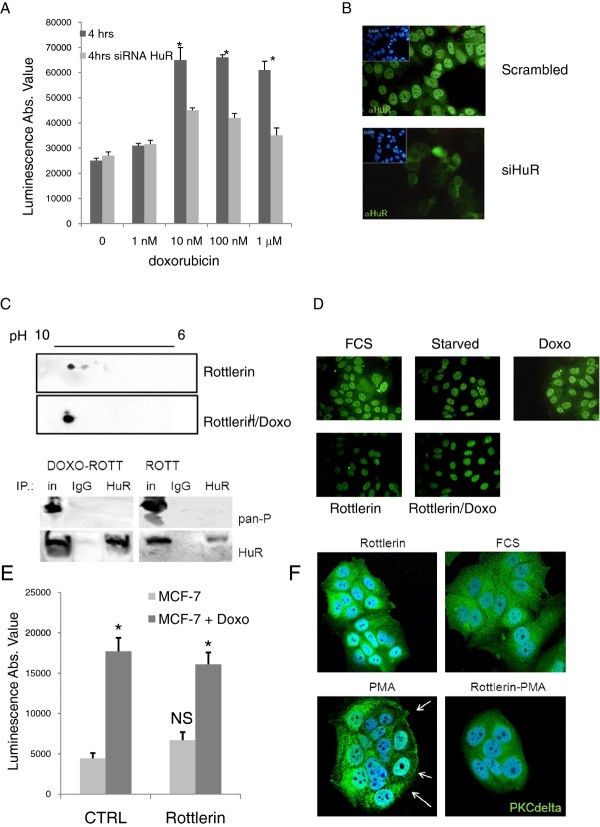
**A: HuR is necessary for caspase 3/7 activation doxorubicin induced**. Histogram shows the caspase 3/7 activation (as luciferase intensity) in MCF-7 cells in the presence of increasing doxorubicin concentration with (light grey bar) or without (dark gray bar) HuR silencing. Graph of a representative experiment out of three experiments. Experiments were run in quadruplicate. * p-value < 0.01 with respect to HuR silenced cells. **B: HuR silencing**. Immunofluorescence on MCF-7 cells at 24 h after the siRNA HuR transfection. HuR is represented in green, counterstaining DAPI is blue. **C: Doxorubicin induced HuR phosphorylation is blocked by rottlerin**. 2D gel western blots on whole MCF-7 cell lysates cells treated with Rottlerin 10 mM for four hours and in co-administration with doxo. HuR immunoprecipitation blotted with anti HuR and pan anti-phospo antibodies. As negative control IgG immunoprecipitation was blotted with anti HuR and pan anti-phospo antibodies. In: Input material, IgG: IgG immunoprecipitated material, HuR: anti-HuR immunoprecipitated material. The drug was tested for its capacity to modify HuR phosphorylation status alone or together with doxorubicin (doxo). Control for doxo alone is shown in Figure 1E **D: HuR localization**. representative immunofluorescence images of HuR localization (green) in control conditions (FCS, starvation, doxo) and in the presence of Rottlerin and with pre-treatment with one hour Rottlerin and four hours doxo. Images indicate that Rottlerin blocks doxo induced HuR translocation. **E. Apoptotic response activation of MCF-7**. Histogram shows the caspase 3/7 activation (as luminescent intensity) of MCF-7 cells in the presence of rottlerin (light grey) or rottlerin with doxorubicin (dark gray). Graph of a representative experiment out of three experiments. Experiments were run in quadruplicate. NS, Not Significant with respect to control (CTRL), * *p*-value < 0.01 with respect to doxorubicin treatment. **F. Rottlerin blocks PMA induced PKCδ activation**. representative confocal immunofluorescence images of PKCδ localization (green) in control conditions (FCS, rottlerin, PMA) and in the presence of coadministration of Rottlerin and PMA. Images indicate that Rottlerin blocks PMA induced PKCδ membrane translocation, known mechanism of PKCδ activation.

**Figure 3:**
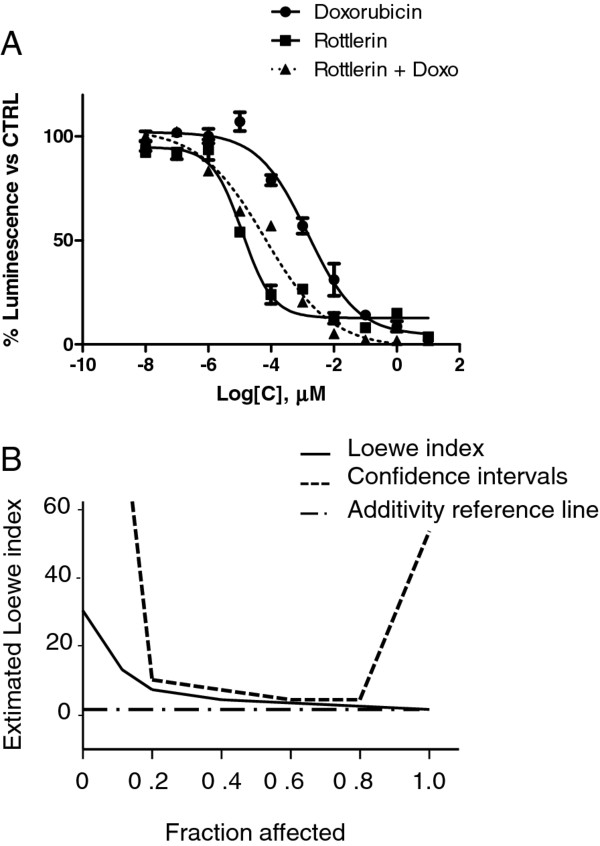
**A. Antagonistic effect of doxorubicin on rottlerin**. Sigmoidal inhibition curves for MCF-7 cells treated either with doxo or rottlerin, or with the two drugs together in a constant ratio of 1:10. The IC_50 _were calculated by nonlinear regression, using Graphpad Prism software. As shown by the dashed line, doxo antagonizes the action of rottlerin, producing a shift to the left of its curve. **B. Graphic representation of the combination index calculated following the Loewe model**. The combination index of the rottlerin-doxorubicin mixture, never goes below the additivity reference line of one, indicating that doxorubicin and rottlerin have antagonistic effects. All the experiments were performed in quadruplicate and repeated at least twice.

We investigated if HuR translocation was involved in doxo-induced cell death. Initially we evaluated the apoptotic response following doxo treatment in the presence and absence of HuR expression in a dose and time dependent manner. The apoptotic response to doxo was measured by the activation of caspase 3 and caspase 7 and by the exposure of phosphatidylserine on the outer leaflet of the plasma membrane (Additional file 1: Figure S1). We transiently transfected MCF-7 cells with a siRNA against HuR (Figure 2B) and found, as shown in Figure 2A, that caspase activation was lower in HuR silenced cells compared to control cells. The decrease of caspase activation was significant after 4 h at 10 nM, 100 nM and 1 μM doxo. We then tested if this effect could be obtained also by blocking doxo-induced HuR phosphorylation by exploiting the known HuR phosphorylation inhibitor rottlerin [28]. Rottlerin administration to starved MCF-7 cells did not influence HuR phosphorylation and slightly influenced the outflow of the protein from the nucleus (Figure 2C, D). However, rottlerin had a strong inhibitory impact on the activation of its first recognized pharmacological target PKCδ (Figure 2F), showing the effectiveness of this drug in this cell line. We measured the apoptotic effect of rottlerin and found that it did not induce an apoptotic response even with a 10 mM dose after a 4 h exposure. Synchronous coadministration of doxo and rottlerin did not increase the apoptotic response with respect to doxo single treatment (Figure 2E). We then preincubated starved cells for 1 h with rottlerin and then added doxo for 4 h. In this condition rottlerin hampered doxo-induced phosphorylation of HuR (Figure 2C) and prevented its cytoplasmic diffusion (Figure 2D). A functional interaction of rottlerin and doxo could be also detected by measuring cell viability, which was determined by an ATP dependent luminescence-based method. Doses of rottlerin and doxo, both separately and in association, ranged from 0.1 nM to 10 μM for a 24 h exposure. The IC50 values in Table 1 show the effect of the administration of the compounds on the proliferation of the MCF-7 cells. Rottlerin exerted an activity in the low nanomolar range, while doxo IC50 was 40 nM, less potent than rottlerin. The combination effect was calculated by the Loewe index, maintaining a fixed concentration ratio of 10:1 between rottlerin and doxo. As shown in Figure 3B, the combination index was significantly above one for the entire fraction of cells affected by the drugs, indicating that the coadministration induced an effect which was less severe than would be expected from the sum of the effects that each drug would produce on its own. One drug, therefore, counteracted some of the effects of the other, thereby behaving as an antagonist.

**Table 1:** IC_50 _Table

	MCF-7		MCF-7/DoxoR		Loewe interaction index		SK-BR-3		SK-BR-3/NOdoxo		MDA-MB-231		MDA-MB-231/DoxoR	
	IC50 (μM)	R sq	IC50 (μM)	R sq	MCF_7	MCF-7/DoxoR	IC50 (μM)	R sq	IC50 (μM)	R sq	IC50 (μM)	R sq	IC50 (μM)	R sq
**Doxorubicin**	0,04	0,96	10	0,96	antagonist	n.c.	10	0,95	50	0,97	5	0,95	75	0,94
**Rottlerin**	0,005	0,96	n.c.	n.c.			n.d.	n.d.	n.d.	n.d.	n.d.	n.d.	n.d.	n.d.

**IC_50 _Table**. IC_50_s of 24 h drug-treated MCF-7 and MCF-7/DoxoR cells. Data obtained were in luminescence units and analyzed with Graphpad Prism software, *n.c*. not convergent, *n.d*. not done.

Taken together, these results show that doxo-induced apoptosis and decrease in cell number depends on the relocalization of HuR in the cytoplasm and is coupled with its phosphorylation.

### HuR binds to target mRNAs and is loaded on polysomes following doxorubicin administration

**Figure 4:**
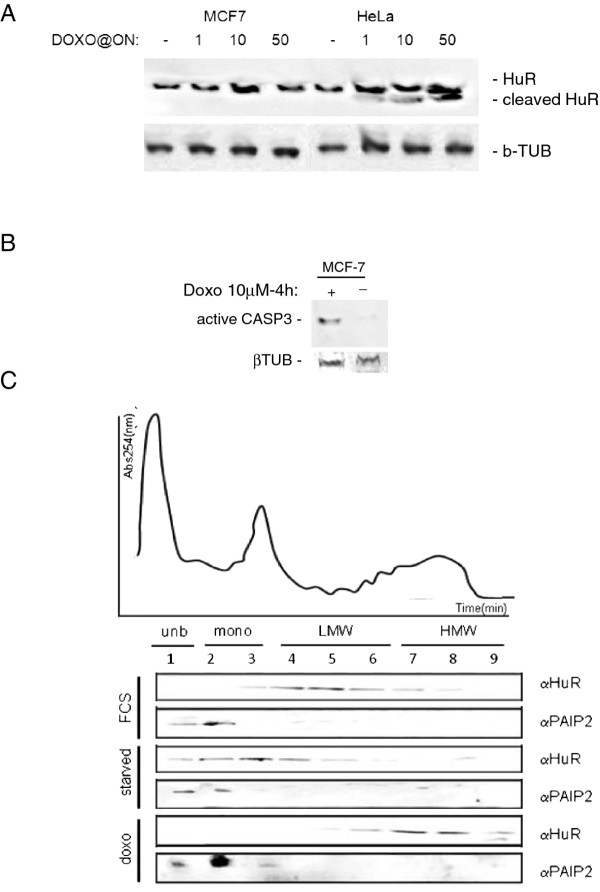
**A: HuR apoptotic cleavage is not present in MCF-7 cells**. Western blotting analysis on HeLa and MCF-7 whole cell lysate sampled after an overnight exposure to doxo at different doses 1, 10, 50 μM (+) or not (-). The cleaved form is present in HeLa cells but only in minimal part in MCF-7 cells. **B: Active caspase 3 is present in MCF-7 cells**. Western blotting analysis on HeLa and MCF-7 whole cell lysate sampled after an overnight exposure to doxorubicine 10 μM (+) or not (-). Active caspase 3 is present in MCF-7 cells. **C: HuR translocates onto the translational active polyribosomes**. Western blotting analysis on polysomal fractionation samples (1-9). Fractionation was performed on MCF-7 cells grown in standard condition (FCS), starved or treated with doxorubicin 10 μM for 4 h and samples were investigated for the presence of HuR or PAIP2. In the upper part of the panel is reported a polysomal profile indicating which fractions are enriched in unbound ribosomes (unb), single ribosomes (mono), low molecular weight polysomes (LMW) or high molecular weight polysomes (HMW). The HMW are the fractions where the translational active polyribosomes sedimentate and the HuR western bloltting signal after doxorubicin treatment localizes. PAIP2 is used as control since localization is not affected.

The dependency of apoptosis on HuR could be ascribed to two previously described mechanisms. One possibility directly favors the aggregation of the apoptosome complex [21] induced by a truncated form obtained following cleavage by caspase 3 and 7. An alternate mechanism relies on an indirect process through post-transcriptional stabilization or increases in the translation of apoptosis related genes [29-32]. We searched for the presence of the cleaved HuR form after doxo in a dose dependent experiment. As shown in Figure 4A, HuR was cleaved minimally and only at 50 μM after overnight exposure in MCF-7 cells. Conversely, HuR was exstensively cleaved, although not completely, in HeLa cells. The presence of both caspases 3 and 7 has been shown to be necessary to cleave HuR [21]. Despite a report about the absence of caspase in MCF-7 cells [33], we and others [34,35] observed the presence of the activated form of the protein following doxo treatment (Figure 4B). HuR is known to localize to polysomes and in stress granules after certain types of stimuli and cell lesions [36,37]. We observed a massive shifting of the protein to heavier polysomal fractions following doxo treatment (Figure 4C), indicating that the protein is actively participating in the cellular response to the drug possibly regulating the translation activity of bound mRNAs.

**Figure 5:**
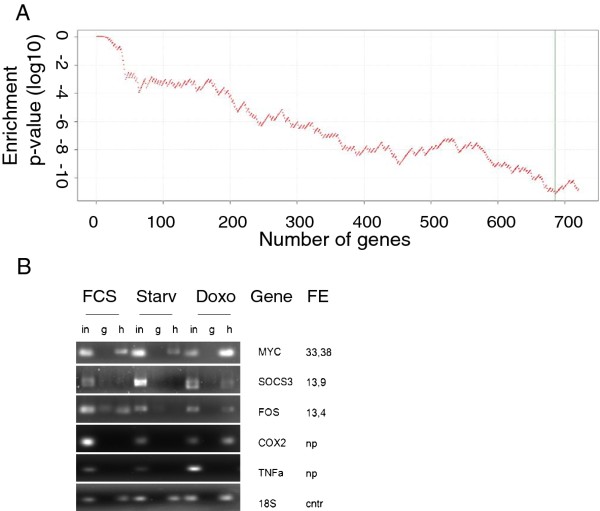
**A: Overepresentation analysis of ARE motif in 3'UTR of HuR bound mRNAs**. The occurrence of AU rich elements (ARE) was annotated on the 3'UTR of all human genes using Transterm. Enrichment analysis of ARE motifs was carried out with a modified Fisher test on the HuR bound genes against the human genome as background. The 721 genes showing significant and specific fold enrichment upon HuR RIP-Chip were sorted in descending order according to their fold enrichment value. For each n from 1 to 721, the first n genes were considered in the enrichment analysis and the corresponding enrichment p-value was calculated and shown in the figure, where the x-axis represents the number of considered genes n, while the y-axis represents the log10 enrichment p-value corresponding to n. The maximum enrichment significance is reached with n = 683, as marked in the figure by a green vertical line. **B: Experimental validation of RIP-Chip**. Semiquantitative RT-PCR on RIP samples immunoprecipitated with anti-HuR antibody (h), whole serum IgG as a negative control (g) and the immunoprecipitation input (in). RIP analysis was performed on MCF-7 cells grown in standard condition (FCS), starved for 24 h (starved) or treated with doxorubicin 10 μM after starvation. Primers to detect mRNAs that showed an high fold enrichment score (MYC, SOCS3, FOS) during RIP-Chip analysis were chosen to test the quality of the microarray read out. Primers to detect COX2 and TNFa are literature reported HuR binding mRNAs and the 18S is a loading and immunoprecipitation control.

To explore the HuR response to doxo in terms of HuR targets, we employed a RIP-chip assay to identify which mRNAs bind to HuR following doxo treatment. After immunoprecipitation and hybridization on Agilent arrays, through a fold enrichment threshold, we filtered those mRNA species specifically bound to HuR. We identified mRNAs corresponding to 822 HGNC annotated genes (721 genes with an annotated 3'UTR, Additional file 2: Table S1) that are bound to HuR and proportionally enriched in the coimmunoprecipitated material. To evaluate the reliability of our procedure of mRNA enrichment, we submitted the 721 HuR-bound gene list to analysis of functional motifs on the 3'UTR. Since HuR is known to bind to AREs, we expected to find a strong overrepresentation of ARE consensus binding in the 3'UTRs of these genes. The enrichment of the ARE was high (28% ARE containing genes, p-value 10^-11^, Figure 5A) and the significance is maintained even when removing progressively genes with lower fold enrichment. The first 50 genes are maximally enriched in ARE (40% of ARE consensus, Table 2). From this list we chose three genes to confirm the result of the genome wide analysis by checking the associated amount on the immunoprecipitation product via semiquantitative PCR of c-fos [38], c-myc [39] and SOCS3. As shown in Figure 5B, all mRNAs confirmed their interaction with HuR after doxo administration.

**Table 2:** List of mRNAs bound to HuR

Gene symbol	HuR fold enrichment	AU-rich stability element (ARE)
**ZNF184**	39.636307	0
**C7orf38**	36.77381	0
**ZNF14**	36.34539	0
**MYC**	33.380974	0
**KLF10**	31.022799	0
**ZNF624**	30.735386	0
**KIAA1383**	27.78753	0
**WDR5B**	24.241896	0
**ZNF443**	24.03738	0
**ZNF606**	23.884226	0
**EGR3**	22.023645	0
**ZNF750**	21.599466	0
**ZBTB6**	21.308832	1
**ZNF367**	20.524889	2
**ZNF44**	18.437605	0
**ZNF658**	18.434893	0
**NFKBIA**	17.987656	1
**FEM1C**	17.382252	0
**FAM5B**	17.253944	1
**TRAF6**	17.018587	0
**ZNF319**	16.993372	0
**NUAK2**	16.958181	4
**ZNF564**	16.866674	0
**EXOC8**	16.58628	3
**HIST1H4B**	16.585716	0
**ZNF217**	16.54123	1
**ZBED5**	16.376629	0
**PNRC1**	16.23357	0
**THAP6**	16.157799	1
**PTGER4**	16.002222	0
**ZIC5**	15.810935	1
**TRIB1**	15.656507	0
**KIAA1586**	15.557861	0
**PLEKHF2**	14.738077	0
**ZNF643**	14.671463	0
**ZNF267**	14.121434	1
**EGR1**	13.995536	0
**ZNF709**	13.961123	1
**SOCS3**	13.905058	2
**TIPARP**	13.888878	4
**CDCA4**	13.859092	1
**FOS**	13.489109	2
**ZNF764**	13.478119	4
**PLK2**	13.028596	2
**DLC1**	12.873251	0
**ELF2**	12.868533	0
**ZNF773**	12.810777	0
**PPP1R3B**	12.796747	0
**SOCS6**	12.379617	1
**NRIP1**	12.327525	0

**List of mRNAs bound to HuR: **first 50 genes, ordered by fold enrichment, identified by RIP-Chip of HuR in the presence of doxorubicin.

These data indicate an involvement of HuR in the mechanism of apoptosis activation induced by doxo in our cellular model system that cannot be explained by the formation of the apoptogenic truncated form of HuR, and that instead HuR is bound to specific mRNAs and relocalizes on polysomes after doxo treatment.

### HuR downregulation mediates doxorubicin drug resistance

**Figure 6:**
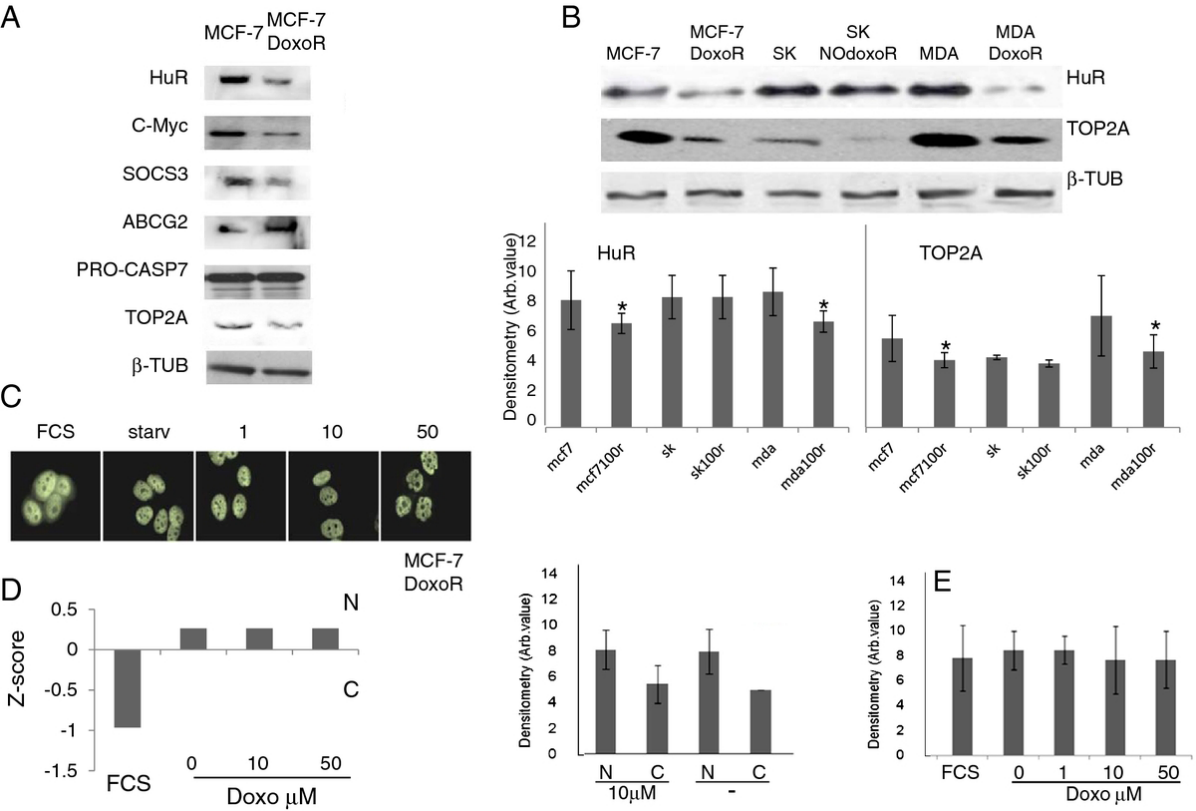
**A. Characterization of MCF-7/DoxoR cells**. Western blotting on whole cell lysates from parental (MCF-7) and doxorubicin resistant (MCF-7/DoxoR) MCF-7 cells. HuR, c-Myc, Socs3 and TOP2A are down regulated in the MCF-7/DoxoR cells in comparison with the parental, ABCG2 is up regulated. Caspase 7 expression level is not affected. Beta tubulin is the loading control. **B**. **Characterization of MDA-MB-231/DoxoR and SK/NOdoxoR cells**. Western blotting on whole cell lysates from parental (MDA-MB-231, SK) and doxo resistant (MDA/DoxoR) and doxo exposed (SK/NOdoxoR) cells. HuR and TOP2A are down regulated in the MDA-MB-231/DoxoR cells. Beta tubulin is the loading control. Protein quantification measured by densitometric analyses on three independent western blots. * p-value < 0.05 with respect to starved conditions. **C**. **HuR does not translocate from the nucleus to the cytosol after doxorubicin treatment in MCF-7/DoxoR cells**. Immunofluorescence (HuR in green, counterstaining DAPI in blue) on MCF-7 cells starved for 24 h and treated at increasing doxo concentration (from 1 to 50 μM) for 4 h. Quantification of HuR cytoplasmic translocation measured on immunofluorescence images by calculating the ratio of intensity signal in the nucleus and in the cytoplasm. **D**. An average of 300 cells for each experimental conditions were used. Z-score below zero indicates nuclear localization, above zero cytoplasmic localization. Quantification of HuR protein translocation after 10 μM doxo for 4 h measured by densitometric analyses on three independent western blots. **E**. Quantification of total HuR protein measured by densitometric analyses on three independent western blots. No change was observed neither in protein amount nor in protein translocation during doxo treatment.

Since HuR downregulation promotes a decrease of the apoptotic response induced by doxo and since rottlerin antagonizes doxo in inducing loss of viability, we wondered if HuR may be implicated in the onset of doxo resistance. We put MCF-7 cells under doxo selection by constantly increasing the drug concentration from 0 to 100 nM in a month time scale. We obtained a cell population, called MCF-7/doxoR, that showed approximately 250-fold resistance to doxo, compared to the wild type MCF-7 cells, as observed by the IC50 increase to approximately 10 μM (Table 1). Further confirmation of the acquired resistance phenotype came from the overexpression in MCF-7/doxoR of the ABCG2 transporter, a typical marker and known cause of doxo pharmacoresistance, while the permissivity to apoptosis was ascertained by caspase 7 expression (Figure 6A). We observed a strong downregulation of HuR as the cells adapted to the presence of doxo. Since we were working on populations, intrinsically subjected to variability, we repeated the procedure of doxo selection three times always obtaining the same clear HuR downregulation. Moreover, we put under selection other two breast cancer cell lines with different charachteristics from MCF-7 cells: MDA-MB-231, triple negative cells, and SK-BR-3, Her2 positive cells. We obtained a population of MDA-MB-231 cells resistant to doxo (MDA-MB-231/doxoR) but not a population of SK-BR-3 (SK-BR/NOdoxoR) according to the IC50 values measured (Table 1). Interestingly, we observed HuR downregulation in MDA-MB-231/doxoR but not in SK-BR-3/NOdoxoR (Figure 6B), suggesting that breast cancer cells downregulate HuR expression only when a deep genetic reprogramming towards pharmacoresistance is taking place and not as a consequence of the mere presence of doxo. Therefore, we investigated if HuR downregulation would have an impact on the levels of bound mRNAs and consequently on their corresponding proteins. We choose c-Myc and SOCS3, as HuR targets, and observed their decrease in concomitance to HuR reduction in MCF-7/doxoR (Figure 6A). Moreover HuR cellular localization was affected in MCF-7/doxoR since the protein was less readily distributed in the cytoplasm after doxo administration, indicating that alterations of the functionality of those pathways that trigger HuR translocation occurred within this cell line during the insurgence of pharmacoresistance (Figure 6C) while its expression level remained unchanged (Figure 6E). We also investigated the expression level of topoisomerase 2A (TOP2A), since its downregulation is a possible mechanism of doxo resistance [9] and since it has been very recently demonstrated that its mRNA is post-transcriptionally regulated by HuR [40]. Indeed, TOP2A protein levels were significantly decreased in MCF-7/DoxoR and MDA-MB-231/DoxoR cells with respect to wild type populations but not in SK-BR-3/NOdoxoR (Figure 6A). Although we did not find TOP2A mRNA in our HuR RIP-chip experiment, TOP2A dowregulation could be a consequence of HuR dowregulation and explain the loss of efficacy of doxo.

**Figure 7:**
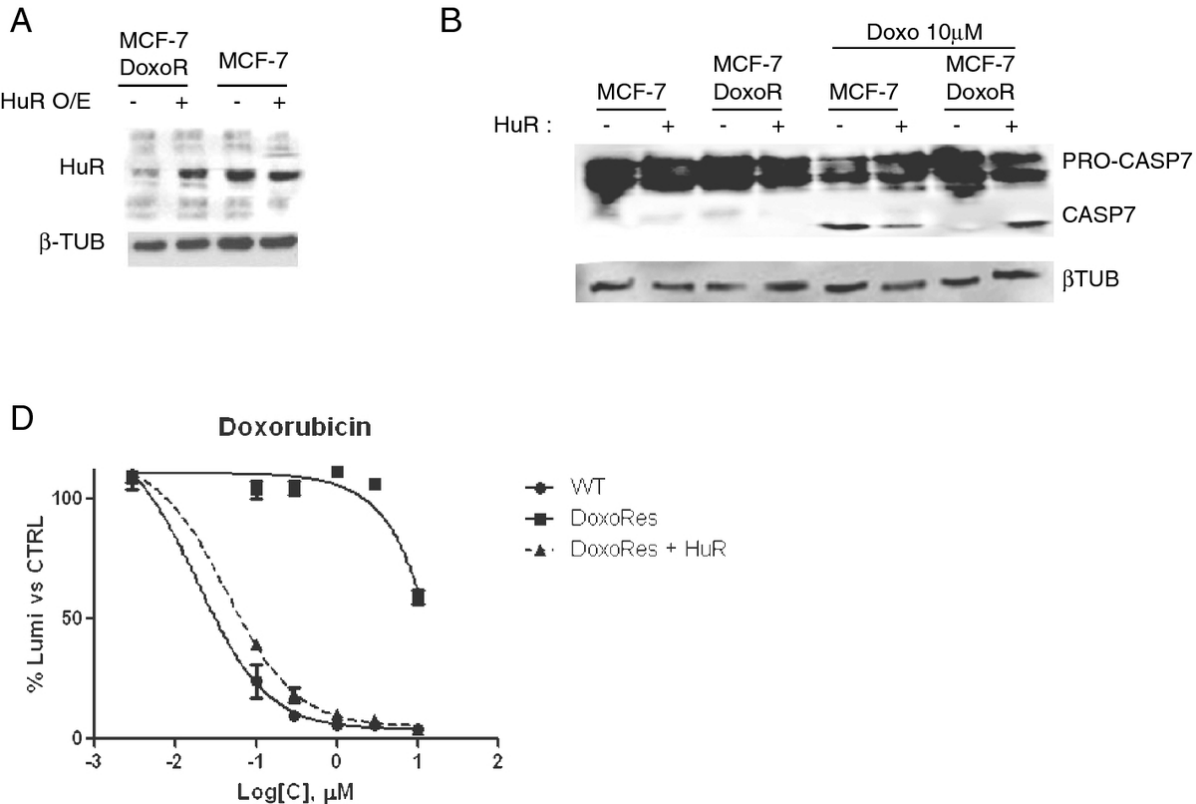
**A: Transfection with HuR overexpressing plasmid**. Western blotting on whole cell lysates from parental (MCF-7) and doxorubicin resistant MCF-7 (MCF-7/DoxorR) cells, mock transfected, to detect the HuR expression level also in the presence (+) of HuR overexpressing plasmid. Beta tubulin was used as loading control. **B. Caspase 7 activation in MCF-7/DoxoR**. Western blotting on whole cell lysates from parental (MCF-7) and doxorubicin resistant MCF-7 (MCF-7/DoxorR), mock transfected, to detect caspase 7 activation in the presence of doxo **C. HuR revert MCF-7 doxorubicin resistance**. Sigmoidal inhibition curves of MCF-7 wild type (WT), doxorubicin-resistant (MCF-7/DoxoR), and MCF-7/DoxoR trasfected with a plasmid containing the coding sequence of HuR (MCF-7/DoxoR + HuR). Cells were exposed to different concentration of doxorubicin for 24 h before being assayed. As demonstrated by the dashed line, the doxo toxic effect on HuR transiently transfected-DoxoRes cells was similar to the one exerted on MCF-7.

In order to evaluate if HuR loss caused the acquired resistance to doxo, we reconstituted HuR expression in the drug-resistant population. Doxo-induced apoptosis, measured by the appearance of the caspase 7, was rescued after 24 h of HuR transfection and in concomitance with HuR overexpression (Figure 7A, B). Finally, to demonstrate the importance of HuR in the acquisition of the resistant phenotype, we measured the toxicity effect of doxo in MCF-7/doxoR transfected with HuR. As can be observed in Figure 7C the dose-response curve of the transfected cells nearly overlaps with the curve obtained with the wild type cells, demonstrating the full reconstitution of the toxic effect of doxo.

Therefore, downregulation of HuR levels and decreased activitation of HuR translocation not only is associated to the acquisition of resistance to doxo but the maintenance of this phenotype is also dependent on the presence of the protein.

## Discussion

In this study we investigated the role of the protein HuR during the cellular response to the chemotherapeutic agent doxo, demonstrating its involvement in doxo-induced apoptosis and in the onset of *in vitro *resistance to this drug in breast cancer cells. We showed that HuR plays a role in modulating gene expression of MCF-7 cells exposed to doxo in a manner similar to what is observed after exposure to other DNA damaging agents [41]. Doxo disrupts the HuR localization equilibrium and thus increases the cytoplasmic concentration of HuR (Figure 1). Indeed, we observed an almost two fold increase in relocalization to the cytoplasm without a relevant change in the overall total protein amount. During HuR relocalization, HuR binds to ARE containing mRNAs (Figure 5B). HuR has been proposed to be an anti-apoptotic protein due to its ability to bind and prolong the stability of anti-apototic genes such as BCL-2 and MCL-1 [32]. On the other side, a direct role for HuR in the molecular processes of apoptosis was first demonstrated by Gallouzi et al. [21] where they showed that, in HeLa cells exposed to staurosporine, the down-regulation of HuR delays apoptosis. In this case, HuR plays an active role in the process, mediated by caspase 3 and 7 cleaving of cytosolic HuR that, after being truncated, helps to promote cell death by binding to pp32. Therefore, HuR probably plays a double role in apoptosis, including an indirect role by positively controlling gene expression of apoptotic genes and a direct role by helping, at the molecular level, the apoptotic machinery to proceed. In our study we demonstrated that in MCF-7 cells HuR is necessary to allow the apoptotic response induced by doxo. When we silenced this gene the response decreased (Figure 2A), but the truncated form of HuR did not appear to be involved in this mechanism since we observed only very low levels of the truncated form after doxo administration (Figure 4A). Therefore, in order to elucidate the role of HuR in regulating apoptosis or pro-survival we used a drug, rottlerin, known to block HuR phosphorylation. This drug was originally identified as a PKCδ inhibitor [42] but, later on, its mechanism of action was correlated to its mitochondrial uncoupler activity [43]. Recently, it has been observed to impair the ability of PKCδ to phosphorylate the Ser318 residue of HuR in colon cancer cells [44]. We observed that rottlerin was able to inhibit also HuR translocation after doxo treatment (Figure 4B). Rottlerin elicited a strong toxic effect on MCF-7 cells without inducing apoptosis. The HuR protein has been described as involved in tumor aggressiveness [45], cancer ethiology [19] and proposed as a potential drug target [46,47] in cancer but, when we coadministered rottlerin and doxo, we observed an antagonistic effect of the two drugs on cell viability. This observation reveals that the two drugs have opposite effects at the molecular level on cellular pathways and is consistent with the opposite effects that the two drugs exert on HuR. Doxorubicin induces apoptosis according to the presence of HuR and accumulated HuR in the cytoplasm, while rottlerin maintained HuR in the nucleus and had a low impact in inducing apoptosis. The observation that HuR is downregulated at the protein level in resistant populations as MCF-7/doxoR and MDA-MB-231/DoxoR but not in cells that did not acquire pharmacoresistance, although exposed to same doses of doxo, as (SK-BR-3/NOdoxoR) cells is in line with its key activity in doxo-induced cytotoxicity. Cells resistant to doxo-induced apoptosis activate the expression of drug extrusion channels, of which we verified ABCG2 as being the major mechanism of drug resistance mediated by the overexpression of detoxifying channels as ABCG2 or ABCB1 [5,6] while the involvement in the process of post-transcriptional regulators, such as HuR, is not widely explored. The activity of HuR has been correlated as a proactive factor in the onset of drug resistance in glioma [48] and against UVR [49]. Moreover in MCF-7 cells cytoplasmic HuR was proposed as a key mediator of tamoxifen resistance, due to its ability to stabilize mRNAs that encode proteins responsible for the activation of the MAPK pathway [50]. Conversely, pancreatic cancer cells overexpressing HuR are more sensitive to gemcitabine compared to control cells [51] due to a stabilization of the deoxycytidine kinase (dCK) mRNA, encoding the enzyme that metabolizes and thereby activates gemcitabine. Very recently Srikantan et al. [40] demonstrated that HuR stabilizes TOP2A mRNA and competes with the microRNA miR-548c-3p, being their combined action a way of controlling TOP2A expression levels and determining the effectiveness of doxo. In our case, we have clear indications that, in the absence of HuR, doxo cannot elicit apoptosis both in MCF-7 wild type cells and in the corresponding doxo-resistant cells. In our MCF-7 and MDA-MB-231 doxo-resistant cells the resistance mechanism could lay on the post-transcriptional regulation of TOP2A, although we did not find TOP2A messenger bound to HuR or downregulated, in the microarray experiment, at the cytoplasmic level. As support to this hypothesis we also found a slower HuR cytoplasmic translocation after doxo administration in MCF-7/DoxoR cells, suggesting that, not only HuR expression level but also the mechanisms activating HuR translocation are altered in resistant cells. The perfect reversion of doxo resistance by HuR re-expression in the experiment of genetic rescue, notwithstanding the permanence of ABCG2 transporter upregulation, further demonstrates the key role exerted by this protein to mediate efficacy of doxorubicin.

## Conclusions

HuR has been correlated in many studies with increased malignancy of tumors, but in this case its expression is a clear indication of the efficacy of doxo treatment. In line with this observation, its downregulation in resistant cells is a determinant of this resistance and therefore its downregulation in cancers treated with doxo could be a marker of pharmacoresistance. In conclusion, although our study was conducted *in vitro *and its generality *in vivo *must be demonstrated, we can suggest taking particular care in the interpretation of HuR expression levels and cell localization in cancer, since its downregulation could be expected to be an indicator of bad prognosis in tumors treated with doxo.

## Methods

### Cell lines

MCF-7, MDA-MB-231, SK-BR-3 breast cancer cell lines where were cultured in complete DMEM (Lonza) supplemented with 10% fetal calf serum (FCS- Lonza), 2 mM L-glutamine, 100 U/ml penicillin, 100 μg/ml streptomycin (Lonza), or OptiMem (Lonza). Doxorubicin resistant cells were derived from the parental cell line by continuously exposing cells to increasing doxorubicin concentration. Doxorubicin was removed from medium 3 days before any experiments were run.

### Chemicals and antibodies

Doxorubicin hydrochloride D1515 Sigma, Anti-HuR sc-71290 santa cruz, Anti-myc 06-340, Millipore normal mouse total serum IgG sc-2025 santa cruz, Anti-c-myc sc 40 santa cruz, anti-SOCS3 sc7009 santa cruz, anti-Caspase 7 sc 56067 santa cruz, anti-beta-tubulin sc 55529 santa cruz, anti-ABCG2 MAB995 R&D, anti-LDH L7016 Sigma, Caspase Glo 3/7 codice prodotto: G8091 Promega, anti-H3 ab1791 Abcam, TransIT-LT1 Transfection Reagent MIR2300 Mirus, HuR siRNA HuR siRNA (h): sc-35619 santa cruz, c-Myc siRNA c-Myc siRNA (h): sc-29226 santa cruz, scrambled control Control siRNA-A sc-37007 santa cruz, anti-active caspase 3 ab13847 Abcam

### Apoptosis assays

MCF-7 or MCF-7/DoxoR cells were seeded in 96-well plates (Corning, Lowell, MA) at a density of 10000 cells/well. The following day, the test drug was added and the cells were exposed to it for 4 h before being assayed using a luminescence-based apoptosis kit (Caspase-Glo^® ^3/7 Assay, Promega, Madison, WI). Statistical analysis was performed using *T*-test algorithm in Xcel (Microsoft) software.

### Plasmid preparation

HuR CDS was PCR amplified from cDNA and blunt inserted in pENTR vector (Gateway system, Invitrogen) using pENTR/SD/D-TOPO cloning system. HuR CDS was then recombined into pT-Rex-DEST30 destination vector for expression in mammalian cells. The cloning procedure was made according to manufacturer instructions. Oligos used for PCR amplification were: Hur-entr-FOR CACC ATGTCTAATGGTTATG AAG ACC AC, Hur-entr-REV TCA TTA TTT GTG GGA CTT GTT GGT TTT G. CDS sequence and orientation into plasmids were verified by sequencing.

### Toxicity assays

MCF-7 or MCF-7/DoxoR cells were seeded in 96-well plates (Corning, Lowell, MA) at a density of 10000 cells/well. The following day, the test drug was added and the cells were exposed to it for 24 h before being assayed using a luminescence-based viability kit (CellTiter-Glo^®^, Promega, Madison, WI). The data were analyzed with GraphPad Prism 5.0 (GraphPad, San Diego, CA) software. The IC50 was determined by fitting the data point with the sigmoidal curve and calculating the dose necessary to achieve half of the maximum effect. The combination index was measured using Mixlow software (version 1.0.0) using dose response curves obtained by mixing Rottlerin and doxo at a fixed ratio of 10:1.

### Immunofluorescence

Cells were plated on acid-washed glass coverslips on plates and maintained in the appropriate culture medium and experimental conditions. In brief, cells were fixed in PHEM buffer (36.8 g/l PIPES, 13 g/l HEPES, 7.6 g/l EGTA, 1.99 g/l MgSO4, titrated to pH 7.0 with KOH) plus 3.7%paraformaldehyde (PFA) for 15 min at room temperature. Cells were then treated for 5 min with HEPES-based permeabilization buffer (300 mM sucrose, 0.2% Triton X-100) and then for 15 min with blocking buffer (3% Bovine Serum Albumin in PBS). Primary antibodies and secondary fluorophore conjugated (Alexa 488) antibodies were diluted in PBS + BSA 0.2%. DAPI (1.5 μg/ml) in PBS + BSA 0.2% was used as counterstaining. Nikon A1R Confocal Laser Microscope, exitation:488 nm and 405 nm 60× APO Oil objective was used for imaging. Cells for fluorescence quantification of the nucleus-cytosol translocation were imaged using an Zeiss 40× LD Plan-*Neofluar 40x*/0.60 on a Zeiss Axio observer Z1, excitation 360/40 or 490/20. Images were processed by Columbus Software (Perkin Elmer) and nucleus-cytosol translocation was expressed in z-score (z = x-μ/δwere μ and δ are respectively the mean and the standard deviation of the whole population of signals and x is the mean the single experimental point) of the ratio: nucleus florescence/cytosol fluorescence, analyzing 300 cells for each experimental point.

### 2D gel electrophoresis

About 250-400 μg of protein from total extracts were added to 180 μl rehydration buffer (8 M urea, 2% CHAPS, 20 mM dithioerythritol, 0.8% IPG buffer, carrier ampholytes pH 6-11 Linear). Samples were applied onto ceramic strip holders (GE) connecting two electrodes, in contact with polyacrylamide gel strips (immobiline dry strips GE ph 6-11 7 cm 17-6001-94). Isoelectrofocusing (IEF) was performed on IPGphor (GE) with 2 different protocols according to the manufacturer recommendations. Second dimension electrophoresis was performed using a Protean II apparatus (Bio-Rad). Strips were soaked first in Equilibration buffer (EB: 6 M urea, 3% SDS, 375 mM Tris pH 8.6, 30% glycerol, 2% DTE), then in EB containing 3% iodoacetamide (IAA) and traces of bromophenol blue (BBP). Subsequently, strips were applied onto 10%-12% PA gels and western blotted.

### RNA immuneprecipitation (RIP)

12 × 10^6 ^MCF-7 cells cultured in the different experimental conditions were syringed by an U-100 insulin needle in 500 ul lyses NT2 buffer (50 mM tirs-HCl pH7.7, 150 mM NaCl, 1 mM MgCl2; 0.05%NP40, 1 U/ul RNase IN, 20 mM DTT, 1% BSA, Protease inhibitor cocktail manufacturer's recommended) chilled at 4°C. Lysate was centrifuged at 10000 g for 10 min then the supernatant was pre-cleared by interaction with protein-A-coated agarose beads (equilibrated in NT2 buffer) for an overnight at 4°C in constant shaking (100 ul slurry beads/500 ul lysate). 150 ul of the pre-cleared lysate were put to interact with protein A coated agarose beads anti-HuR antibody (or control IgG) conjugated for 6 h at 4°C then washed twice in NT2 buffer. 20 ul Protein-A-coated slurry agarose beads were conjugated with 4 ug antibody at room temperature for 2 h, washed and equilibrated in NT2 lysis buffer before use. RNA was isolated from the different samples (immunoprecipitated anti-HuR, IgG and precleared input) by TriZol as manufacturer's recommended, retrotranscribed into cDNA by MBI-Fermentas kit and used as template for PCR analysis. Primers used are FOS F:ATGAGCCTTCCTCTGACTCG, R:ACGCACAGATAAGGTCCTCC. MYC F:GCCACGTCTCCACACATCAG, R:TGGTGCATTTTCGGTTGTTG. SOCS3 F:TATTAGGAGATGCTTGAAGAA, R:ATAGTGCTCTTTATTATAAAT.18S, F:TACCTGGTTGATCCTGCCAGTAGCATA, R:AGGAACCATAACTGATTTAATGAGCCAT, TNF:F, AAGCATGATCCGGGACGTGGAGCTGGCCGA, R:TCTGGGGGCCGATCACTCCAAAGTGCAGCA, COX2F:GTGCGCGGTCCTGGCGCTCAGCCATACAGC, R:AAGGCTTCCCAGCTTTTGTAGCCATAGTCA

### Microarray data analysis

RIP samples and cytosolic RNA samples were labeled using a Quick Amp dual Colour 5190-0444 and hybridized on a Gene expression All Human Genome (4 × 44 K) oligo microarray kit Aglient Thecnology G4112F. Hybridized microarray slides were scanned with an Agilent DNA Microarray Scanner (G2505C, Agilent Technologies, Santa Clara, CA) at 5 micron resolution with the manufacturer's software (Agilent ScanControl 8.1.3). The scanned TIFF images were analyzed numerically using the Agilent Feature Extraction Software version 10.7.7.1 according to the Agilent standard protocol GE1_107_Sep09. Following analyses were carried with GeneSpring GX 9 software. All microarray data are available through the Gene Expression Omnibus database http://www.ncbi.nlm.nih.gov/geo/ using the accession number GSE33055.

#### Comparison between cytoplasmic RNA samples of control MCF7 cells with doxorubicin treated cells

Experiments were conducted in biological quadruplicate. Microarray signals were log2 transformed, normalized using 75th percentile-shift and baseline transformed to the median of all samples. Probes flagged as absent in all samples were removed. Probes with high coefficient of variation (> 50%) between replicas of the same condition were removed. Differentially expressed genes were detected applying a significance threshold on *t*-test unequal variance (Benjamini-Hochberg corrected p-value < 0.05) and a fold change threshold (absolute fold change > 2).

#### Comparison between HuR RIP samples and IgG RIP samples of doxorubicin treated cells

Experiments were conducted in biological quadruplicate. Microarray signals were log2 transformed. Normalization and baseline transformation were not applied. Probes flagged as absent in all samples were removed. Probes with high coefficient of variation (> 50%) between replicas of the same condition were removed. Differentially expressed genes (specifically bound by HuR) were detected applying a significance threshold on *t*-test unequal variance (Benjamini-Hochberg corrected p-value < 0.05) and a fold change threshold (positive fold change > 2).

#### Comparison between HuR RIP samples and cytoplasmic RNA samples of doxorubicin treated MCF7 cells

Experiments were conducted in biological triplicate. Microarray signals were log2 transformed, normalized using 75th percentile-shift and baseline transformed to the median of all samples. Probes flagged as absent in all samples were removed. Probes with high coefficient of variation (> 50%) between replicas of the same condition were removed. Differentially expressed genes were detected applying a significance threshold on *t*-test unequal variance (Benjamini-Hochberg corrected p-value < 0.05) and a fold enrichment threshold (absolute fold change > 2).

#### Ontological enrichment analysis

The DAVID [52] resource was used for gene-annotation enrichment analysis of DEG lists with categories from the following resources: PIR http://pir.georgetown.edu/, Gene Ontology http://www.geneontology.org, KEGG http://www.genome.jp/kegg/ and Biocarta http://www.biocarta.com/default.aspx pathway databases, PFAM http://pfam.sanger.ac.uk/ and COG http://www.ncbi.nlm.nih.gov/COG/ databases. The significance of overrepresentation was determined at a false discovery rate of 5% with Benjamini multiple testing correction.

#### Analysis of 3' UTRs

Human 3' UTR sequences of human genes represented on the Agilent array were downloaded from the UCSC genome browser http://genome.ucsc.edu/, assembly GRC37/hg19. For each HGNC gene a single 3' UTR sequence was determined as the longest among all the gene transcript variants. AU rich elements were mapped to 3'UTR sequences using the Transterm ARE pattern "UAUUUAUWW" [53]http://uther.otago.ac.nz/. Motif enrichment analyses were implemented in R: motif enrichment was assessed calculating the EASE Score [54], a modified Fisher Exact P-Value introduced by DAVID developers http://david.abcc.ncifcrf.gov/home.jsp. In all enrichment analyses, the 14678 human genes with 3' UTR longer than 9 nucleotides were used as background set.

No ethics committee approval has been requested as the research has been entirely performed with commercial cell lines.

## Abbreviations

3'UTR: 3' UnTraslated region; ARE: AU rich element; BP: Biological process; DEGs: Differentially expressed genes; Doxorubicin: Doxo; HCS: High content screening; IgG: Immunoglobulin type G; GO: Gene ontology; KI: Kinase inhibitor; MDR: MultiDrug resistance; PCR: Polymerase chain reaction; RBP: RNA binding protein; RIP: RibonucleoProtein immunoprecipitation; UVR: Ultraviolet radiation.

## Competing interests

The authors declare that they have no competing interests.

## Authors' contributions

EL generated the breast cancer DoxoR cell lines and carried out the immunofluorescence, RIP-Chip, cell fractionation, western blotting, silencing and caspase activation experiments, and performed the cell toxicity and combination drug synergy experiments. TT carried out the RIP-Chip and GE microarray data analysis. GV participated together with EL in the polysomal HuR translocation experiment and discussed data. AMS performed the cell toxicity and combination drug synergy test. EL discussed the experiments and drafted the manuscript. AQ discussed the experiments and participated in critically revising the manuscript. AP conceived study, designed the experiments, supervised the work and wrote the manuscript. All authors read and approved the manuscript.

## Supplementary Material

Additional file 1 **Figure S1**. Doxorubicin induced apoptosis. Annexin-V FACS assay on MCF-7 cells treated with different doxorubicin (doxo) concentration for 18 h or not (untreated). The upper left dot plot indicates which area is occupied by necrotic, apoptotic or living cells respectively. Freeze and tow (F&T) sample was used as necrosis positive control and cycloexamide 10 μM (CXM) was used as apoptosis positive control. Click here for file

Additional file 2 **Table S1**. List of mRNAs bound to HuR in the presence of doxorubicin. The complete list of mRNAs precipitated with HuR during RIP-Chip. Probe indicates the Agilent reference number, gene symbol is the HGNC symbol, HuR fold enrichment is the fold enrichment of mRNA bound to HuR in comparison to cytoplasmic amount, AU-rich stability element is how many AREs are present in the 3'UTR of the mRNA according to TRANSTERM, description is gene annotation. Click here for file
