# Supplementary figures and images for: Downregulation of HuR as a new mechanism of doxorubicin resistance in breast cancer cells
Source: Mol Cancer. 2012 Mar 21;11:13. doi: 10.1186/1476-4598-11-13 (PMC3325864; doi:10.1186/1476-4598-11-13)

## Supplementary figure 1

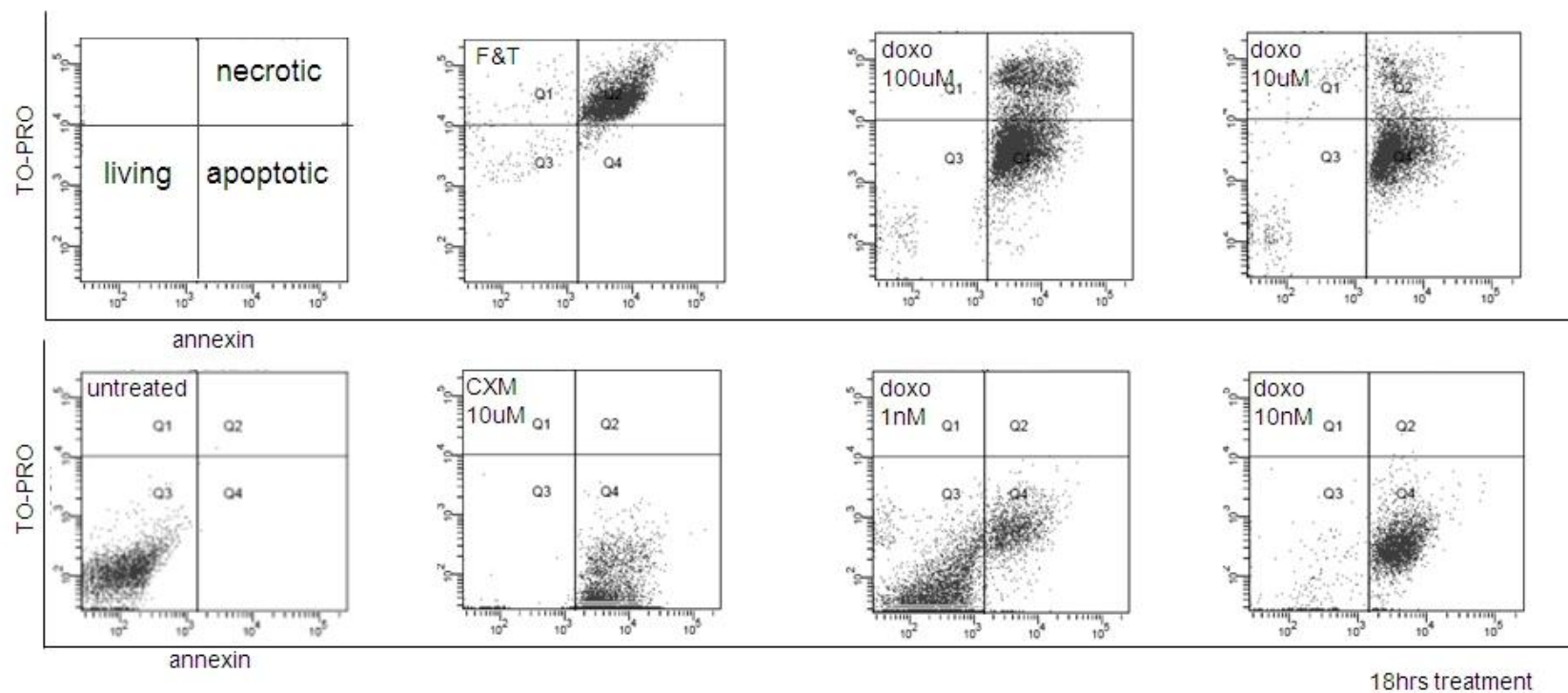

Supplement: Additional file 1 — Figure S1. Doxorubicin induced apoptosis. Annexin-V FACS assay on MCF-7 cells treated with different doxorubicin (doxo) concentration for 18 h or not (untreated). The upper left dot plot indicates which area is occupied by necrotic, apoptotic or living cells respectively. Freeze and tow (F&T) sample was used as necrosis positive control and cycloexamide 10 μM (CXM) was used as apoptosis positive control. [file 1476-4598-11-13-S1.PDF]
